# ﻿Two new acremonium-like species, *Paragibellulopsissinensis* sp. nov. and *Phialoparvumsinense* sp. nov. (Sordariomycetes, Ascomycota) from China

**DOI:** 10.3897/mycokeys.118.155316

**Published:** 2025-06-06

**Authors:** Heng Pan, Shuo-Qiu Tong, Zeng Chen, Yun-Jie Wu, Yi Wang, Gang Tao, Zhi-Yuan Zhang

**Affiliations:** 1 College of Eco-Environmental Engineering, Guizhou Minzu University, Guiyang 550025, China Guizhou Minzu University Guiyang China; 2 College of Life Sciences, Institute of Agro-bioengineering, Guizhou University, Guiyang 550025, China Guizhou University Guiyang China; 3 Nanhu Park, Changchun 130012, China Nanhu Park Changchun China

**Keywords:** Acremonium-like species, fungal classification, new taxa, plectosphaerellaceous fungi, urban soil

## Abstract

*Paragibellulopsis* and *Phialoparvum*, both erected in 2018, currently comprise merely one and three validly published species, respectively. The identification of two new species, *Paragibellulopsissinensis* and *Phialoparvumsinense*, was achieved by analyzing morphological characteristics and phylogenetic data obtained from four molecular markers (ITS, LSU, *rpb2*, and *tef-1α*). Morphologically, *Pa.sinensis* possessed short conidiogenous cells and aseptate and smaller conidia. On the other hand, *Ph.sinense* had obovate conidia and longer phialides. Phylogenetic analysis revealed that *Pa.sinensis* formed an independent branch as a sister species to *Pa.chrysanthemi*, whereas *Ph.sinense* clustered in a monophyletic clade with a stable topology. The maximum likelihood bootstrap ratio and the Bayesian inference posterior probability provided robust statistical evidence, indicating the presence of two novel species within the genera of *Paragibellulopsis* and *Phialoparvum*. The present study contributes to the discovery of species diversity in *Paragibellulopsis* and *Phialoparvum*.

## ﻿Introduction

*Acremonium* has long been recognized as one of the most simply structured fungi of all filamentous asexual fungi (Hou et al. 2023) and referred to the family Bionectriaceae (Hyde et al. 2024). The main characteristics of *Acremonium* are the production of septate hyphae giving rise to narrow, tapered, mostly lateral phialides with unicellular conidia arranged in mucoid heads or unconnected chains, and differentiated conidiophores with or without verticillate branches may be observed in some species (Gams 1971, 1975; Domsch et al. 2007; Perdomo et al. 2011; Summerbell et al. 2011). Although the asexual morph of *Acremonium* is relatively plesiomorphic in morphology, the genus is heterogeneous due to the fact that numerous sexually reproducing ascomycetous genera with diverse morphologies possess acremonium-like asexual morphs.

Recently, Hou et al. (2023), through extensive taxonomic sampling within Hypocreales and multilocus phylogenetic analyses, further confirmed the highly polyphyletic nature of acremonium-like taxa and demonstrated that these taxa are widely distributed across multiple families, including Cephalothecaceae, Chrysonectriaceae, Clavicipitaceae, Cordycipitaceae, Myrotheciomycetaceae, Nectriaceae, Neoacremoniaceae, Niessliaceae, Nothoacremoniaceae, Plectosphaerellaceae, Pseudoniessliaceae, and Valsonectriaceae. This finding further demonstrates that due to the morphological plasticity of acremonium-like species, clear taxonomic delineation of these fungi remains unattainable without DNA barcoding and phylogenetic analyses. Consequently, researchers must integrate DNA sequence data to substantiate morphological and ecological interpretations (Verkley et al. 2013; Chen et al. 2015; Crous et al. 2021).

Zare et al. (2007) established the family Plectosphaerellaceae based on its close relationships to Glomerellaceae within Glomerellales when revising verticillium-like taxa. Giraldo and Crous (2019) comprehensively clarified the systematic status of members of Plectosphaerellaceae based on multi-locus phylogenetic analyses and morphological evidence. Recently, Crous et al. (2023b) proposed the reduction of Plectosphaerellaceae under Trichosphaeriaceae and its elevation to the order Trichosphaeriales, based on a single fungal isolate. However, the type species of two recently described genera, *Parafuscohypha* (only *Para. proliferate*, ex-type CBS 308.74) and *Truncatascus* (only *T.microsporus*, ex-type GZAAS 19-1744), exhibited almost identical ITS and *tef-1α* sequences to a putative *Trichosphaeriapilosa* isolate (CBS 149698, which was designated epitype), suggesting these genera may represent synonyms unless the results were due to contamination (Yeh et al. 2024). These taxonomic changes, based on a single ascomata isolate, are particularly problematic due to potential misidentification or contamination (Crous et al. 2023b). Moreover, the designation of epitype under such circumstances proves superfluous, contravenes established taxonomic protocols, and potentially leads to misleading conclusions (as in *Trichosphaeria*) (Yeh et al. 2024). In light of these concerns, Giraldo et al. (2019) and Yeh et al. (2024) employ the term “plectosphaerellaceous fungi” to replace potentially ephemeral discrete taxonomic names.

In the present study, we aimed to isolate microfungi from urban soil samples in Beijing and Guizhou, China. This study has the following objectives: 1) to describe two novel acremonium-like species (affiliated with the genera *Paragibellulopsis* and *Phialoparvum*) through comprehensive morphological examinations and phylogenetic analyses of ITS, LSU, *rpb2*, and *tef-1α* sequence data; 2) to provide a checklist that includes substrate, availability of molecular data, morphological characteristics, and country of origin.

## ﻿Materials and methods

### ﻿Fungal isolation and morphological characterization

Samples were collected from the green belts of Guizhou Wildlife Park and Beijing Xishan National Forest Park on 17 July 2022 and 4 June 2024, respectively. The samples were collected 3–10 cm below the soil surface, were stored in Ziploc plastic bags, transported to the laboratory, and promptly processed. Fungi were isolated and purified using the method described by Zhang et al. (2023, 2024).

Colony characters and diameters were recorded from cultures grown on potato dextrose agar (PDA), synthetic low-nutrient agar (SNA), and oatmeal agar (OA) plates at 25 °C in the dark for 14 days. For light microscopic observations, slides were prepared from cultures grown on PDA, and the characterization and measurement of fungal microscopic characteristics were performed in 25% clear lactic acid. Other microscopic examinations followed procedures outlined by Senanayake et al. (2020). Fungal structures were photographed using a Nikon ECLIPSE Ni compound microscope fitted with a Nikon DS-Ri2 digital camera. Measurements for all structural components were made with Tarosoft Image FrameWork software (IFW).

Descriptions of novelties were deposited in MycoBank, and the type strains for the novel taxa represented by living cultures were deposited at the China General Microbiological Culture Collection Center (CGMCC), China. The holotype (dried cultures) of the novel species was deposited at the Fungarium (HMAS), Institute of Microbiology, Chinese Academy of Sciences (CAS). In addition, all living cultures were stored in a metabolically inactive state (i.e., kept in sterile 30% glycerol in a –80 °C freezer), which were deposited in the College of Eco-Environmental Engineering, Guizhou Minzu University, China.

### ﻿DNA extraction, PCR amplification, and sequencing

The BioTeke Fungal Genomic DNA Extraction Kit (DP2032, BioTeke, Beijing, China) was used to extract genomic DNA from fungal hyphae according to the manufacturer’s instructions. Four gene regions, internal transcribed spacers (ITS), large subunit rDNA (LSU), transcription elongation factor 1-alpha gene region (*tef-1α*), and RNA polymerase II subunit (*rpb2*), were amplified and sequenced using primers listed in Table 1. Polymerase chain reactions (PCR) were carried out in a 25 μL reaction volume, which contained 12.5 μL 2 × PCR Master Mix (Sangon Biotech, China), 8.5 μL ddH_2_O, 1 μL of each primer, and 2 μL DNA template. All newly generated sequences have been deposited in the GenBank database (Table 2).

**Table 1.** Sequences of primers used in this study.

Gene/loci	PCR primers (forward/reverse)	Primer sequence (5´-3´)	Reference
ITS	ITS1	TCCGTAGGTGAACCTGCGG	White et al. (1990)
	ITS4	TCCTCCGCTTATTGATATGC	White et al. (1990)
LSU	LR0R	ACCCGCTGAACTTAAGC	Vilgalys and Hester (1990)
	LR5	ATCCTGAGGGAAACTTC	Vilgalys and Hester (1990)
*tef-1α*	EF1-983F	GCYCCYGGHCAYCGTGAYTTYAT	Rehner and Buckley (2005)
	EF1-2218R	ATGACACCRACRGCRACRGTYTG	Rehner and Buckley (2005)
*rpb2*	fRPB2-5F	GAYGAYMGWGATCAYTTYGG	Liu et al. (1999)
	RPB2-7cR	CCCATRGCTTGYTTRCCCAT	Liu et al. (1999)

**Table 2.** Strains used in this study, with information on the GenBank accessions of the sequences.

Species	Strains	GenBank accession no.			
		ITS	LSU	*rpb2*	*tef-1α*
*Acremoniisimulanshongheensis*	HKAS 122669 T	OQ379005	OQ379416	OQ378988	OQ378995
*Acremoniisimulanshongheensis*	HKAS 122670	OQ379006	OQ379417	OQ378989	OQ378996
*Acrostalagmusannulatus*	CBS 121.84	LR026673	LR025802	LR026104	LR026374
*Acrostalagmusluteoalbus*	CBS 112.16	LR026668	LR025797	LR026101	LR026369
*Allomusicilliumdomschii*	CBS 764.69 T	OQ429497	OQ055408	OQ453889	OQ470787
*Brunneochlamydosporiumnepalense*	CBS 971.72 IT	LR026684	LR025813	LR026112	LR026386
*Brunneochlamydosporiumterrestre*	CBS 112777 T	LR026690	LR025819	LR026118	LR026392
*Brunneomycesbrunnescens*	CBS 559.73 T	LN810520	HQ231966	LR026119	LN810534
*Brunneomyceseuropaeus*	CBS 652.96 T	LN810519	LN810512	LN810528	LN810538
*Chlamydosporiellarestricta*	CBS 178.40 T	LR026693	LR025822	LR026122	LR026395
*Chlamydosporiellarestricta*	CBS 716.88	LR026696	LR025825	LR026125	LR026398
*Chordomycesalbus*	CBS 987.87 T	DQ825970	JX158444	JX158466	JX158400
*Chordomycesantarcticus*	CBS 120045 T	KJ443241	KJ443109	KJ443157	KJ443197
*Furcasterigmiumfurcatum*	CBS 122.42 T	LR026709	LR025838	LR026133	LR026408
*Furcasterigmiumfurcatum*	CBS 116548	LR026712	LR025842	LR026134	LR026409
*Fuscohyphaexpansa*	CBS 103.95	LR026714	LR025844	NA	LR026411
*Fuscohyphaexpansa*	CBS 418.89 T	LR026715	LR025845	LR026136	LR026412
*Gibellulopsisnigrescens*	CBS 120949 NT	LR026738	LR025868	LR026149	LR026429
*Gibellulopsisserrae*	CBS 290.30 T	LR026742	LR025872	NA	LR026433
*Houtenomycescaricicola*	CBS 149675 T	OQ628483	OQ629065	OQ627944	NA
*Lecterahumicola*	IMI 265740 T	JQ647449	LR025896	LR026169	LR026457
*Lecteraphaseoli*	IMI 366179 T	JQ693168	LR025898	LR026171	LR026459
*Longitudinalisnabanheensis*	KUMCC 16-0145 T	KY882037	NG_068250	NA	KY882040
*Musicilliumtheobromae*	CBS 968.72 NT	LR026773	LR025907	LR026178	LR026468
*Musicilliumtropicale*	CBS 120009 T	LR026783	LR025917	LR026186	LR026477
*Musidiumstromaticum*	CBS 135.74A	LR026787	LR025922	LR026189	LR026482
*Musidiumstromaticum*	CBS 863.73 T	DQ825969	HQ232143	NA	LN810533
*Nigrocephalumcollariferum*	CBS 124585	FJ765365	LR025928	LR026192	LR026485
*Nigrocephalumcollariferum*	CBS 124586 T	FJ765367	LR025929	LR026193	LR026486
*Parafuscohyphaproliferata*	CBS 308.74 T	OQ429771	OQ055669	OQ560705	OQ471098
*Paragibellulopsischrysanthemi*	MAFF 242621 T	KC287235	KC287230	NA	KC287232
*Paragibellulopsischrysanthemi*	MAFF 243429	KC287234	KC287229	NA	KC287231
***Paragibellulopsissinensis***	**CGMCC 3.28460 = ZY 24.001 T**	**PQ536128**	**PQ536126**	**PQ540986**	**PQ540988**
***Paragibellulopsissinensis***	**ZY 24.002**	**PQ536129**	**PQ536127**	**PQ540987**	**PQ540989**
*Paramusicilliumasperulatum*	CBS 120158 T	LR026792	LR025930	LR026194	LR026487
*Phaeochloridiumtaiwanense*	BCRC FU31690 T	PP278369	NA	LC799521	LC799522
*Phaeochloridiumtaiwanense*	R. Kirschner 5374	PP278370	NA	LC799524	LC799523
*Phialoparvumbifurcatum*	CBS 299.70B T	LR026793	LR025931	LR026195	LR026488
*Phialoparvummaaspleinense*	CBS 145321 T	LR590190	LR590368	LR594791	NA
*Phialoparvumrietveltiae*	CBS 145322 T	LR590191	LR590367	LR594790	NA
***Phialoparvumsinense***	**CGMCC 3.27537 = ZY 22.078 T**	**PP769719**	**PP769727**	**PP779747**	**PP779739**
***Phialoparvumsinense***	**ZY 22.079**	**PP769720**	**PP769728**	**PP779748**	**PP779740**
***Phialoparvumsinense***	**ZY 22.080**	**PP769721**	**PP769729**	**PP779749**	**PP779741**
***Phialoparvumsinense***	**ZY 22.081**	**PP769722**	**PP769730**	**PP779750**	**PP779742**
*Plectosphaerellacucumerina*	CBS 137.37 T	LR026798	LR025936	LR026199	LR026493
*Plectosphaerellanauculispora*	CGMCC 3.19656 T	MK880441	MK880431	MK930460	MK930453
*Sayamraellasubulata*	BCC 78964 T	LR026833	LR025971	LR026226	LR026531
*Sinochloridiumbambusicola*	CGMCC 3.20735 T	OL628215	NA	NA	NA
*Sodiomycesalcalophilus*	CBS 114.92 IT	JX158421	JX158443	JX158465	JX158399
*Sodiomycesalkalinus*	CBS 110278 T	NR_145378	JX158427	JX158449	JX158383
*Stachylidiumbicolor*	CBS 121802 ET	LR026834	LR025972	NA	LR026532
*Stachylidiumpallidum*	DAOMC 226658	LR026838	GU180651	LR026228	LR026534
*Summerbelliaoligotrophica*	CBS 299.70G	LR026716	LR025846	NA	LR026413
*Summerbelliaoligotrophica*	CBS 657.94 T	LR026719	LR025849	NA	NA
*Theobromiumfuscum*	CBS 112271 T	LR026839	LR025976	LR026229	LR026535
*Trichosphaeriapilosa*	CBS 149698 ET	OQ990134	OQ990085	OQ989224	OQ989249
*Truncatascusmicrosporus*	GZAAS 19-1744 T	OR225088	OP099542	OR146950	NA
*Truncatascusmicrosporus*	GZAAS 21-2018	OR225089	OR209670	OR146951	NA
*Verticilliumalfalfae*	CBS 130603 T	LR026851	LR025988	LR026236	LR026547
*Verticilliumnubilum*	CBS 457.51 T	LR026937	LR026076	LR026282	LR026591
*Xenoplectosphaerellaclematidis*	MFLUCC 17-2067 T	NR_172181	NG_071256	MT394722	MT394674
*Monilochaetesinfuscans*	CBS 379.77	LR026764	GU180645	GU180658	LR026460
*Monilochaetesinfuscans*	CBS 869.66	GU180626	GU180639	GU180657	LR026461

Notes: T: Ex-type; IT: Ex-isotype; NT: Ex-neotype; ET: Ex-epitype; CBS: Westerdijk Fungal Biodiversity Institute, Utrecht, Netherlands; CGMCC: China General Microbiological Culture Collection Center, Beijing, China; HKAS: herbaria of the Kunming Institute of Botany, Chinese Academy of Sciences, Kunming, China; IMI: International Mycological Institute, CABI-Bioscience, Egham, Bakeham Lane, UK; KUMCC: Kunming Institute of Botany Culture Collection, Kunming, China; MAFF: Ministry of Agriculture, Forestry and Fisheries, Ibaraki, Japan; BCRC: Bioresource Collection and Research Center, Taiwan, China; BCC: BIOTEC Culture Collection, Pathumthani, Thailand; DAOMC: Canadian Collection of Fungal Cultures, Ontario, Canadian; GZAAS: Guizhou Academy of Agriculture Sciences, Guizhou, China; MFLUCC: Mae Fah Luang University Culture Collection, Chiang Rai, Thailand; Other acronyms represent personal collections; NA, not available. DNA sequences for the new isolates were in bold.

### ﻿Phylogenetic analyses

Lasergene software (version 6.0, DNASTAR) was used to edit the ambiguous bases of the PCR amplicon sequences. Combined sequences of ITS, LSU, *rpb2*, and *tef-1α* were analyzed to infer the phylogenetic placement of *Pa.sinensis* and *Ph.sinense*. The sequences were aligned and adjusted by MAFFT v7.037 (Katoh and Standley 2013) and MEGA 6.06 (Tamura et al. 2013). The “Concatenate Sequence” function in the PhyloSuite platform version 1.2.3 (Xiang et al. 2023) was used for the concatenation of loci.

The best-fit substitution model was selected for the Bayesian analysis and the maximum likelihood analysis using the corrected Akaike Information Criterion (AICc) in the ModelFinder (Kalyaanamoorthy et al. 2017). The maximum likelihood analysis was implemented in IQ-TREE v1.6.11 (Nguyen et al. 2015) with 10,000 bootstrap tests, using the ultrafast algorithm (Minh et al. 2013), in the PhyloSuite v.1.2.3 (Xiang et al. 2023). MrBayes version 3.2 (Ronquist et al. 2012) was used for the BI analysis. The Markov Chain Monte Carlo (MCMC) method was used to perform 5×10^7^ simulations with a sampling frequency of 10^3^ generations and a 25% burn-in. After the analysis was ﬁnished, Tracer v1.5 (Drummond and Rambaut 2007) was used to determine burn-in and conﬁrm that both runs had converged.

## ﻿Results

### ﻿Phylogenetic analyses

**Figure 1.**
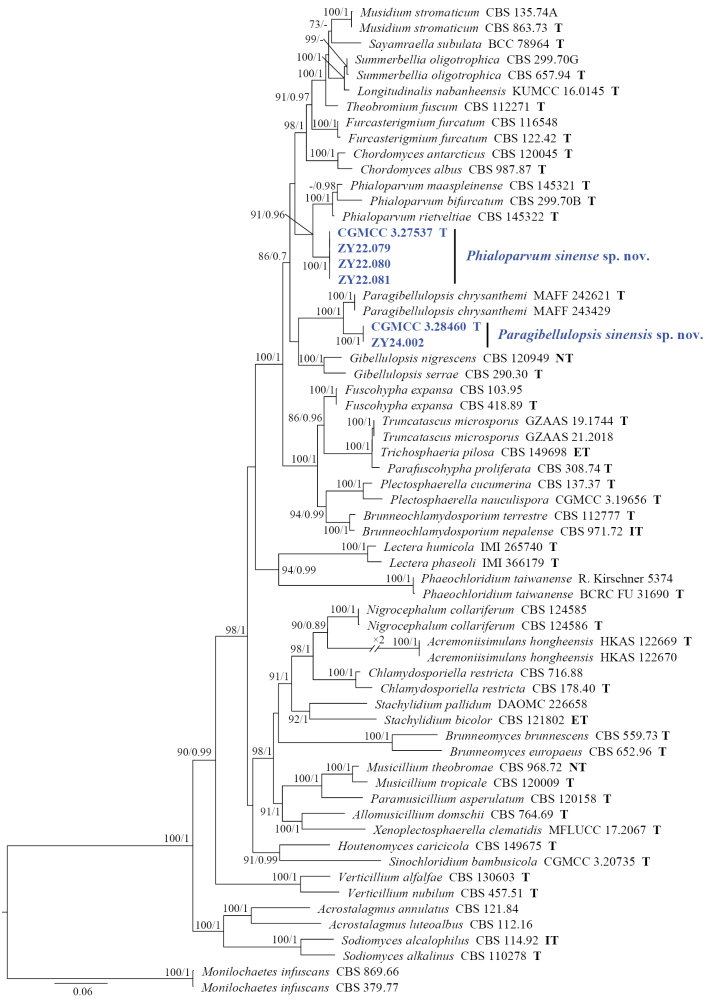
Phylogenetic tree inferred from a maximum likelihood analysis based on a concatenated alignment of ITS, LSU, *rpb2*, and *tef-1α* sequences of 63 strains representing plectosphaerellaceous fungi and outgroups. The ML bootstrap support values (MLBS) above 70% and Bayesian posterior probabilities (BPP) above 0.70 are given at the nodes (MLBS/BPP). Strains with special status are indicated with a superscript letter after the accession number (T: ex-type, ET: ex-epitype, IT: ex-isotype, NT: ex-neotype). The new species are printed in blue font. The tree is rooted to *Monilochaetesinfuscans*CBS 869.66 and CBS 379.77.

Based on the combined dataset of the ITS, LSU, *rpb2*, and *tef-1α* sequences, the phylogeny of *Pa.sinensis*, *Ph.sinense*, and its related taxa was studied. *Monilochaetesinfuscans* (CBS 869.66 and CBS 379.77) were used as the outgroup for the phylogenetic analysis. The concatenated dataset included 63 taxa and consisted of 2,823 nucleotides (ITS, 499 bp; LSU, 795 bp; *rpb2*, 743 bp; and *tef-1α*, 786 bp) with inserted gaps. The phylogenetic tree of the multi-locus dataset revealed that all genera were grouped in distinct branches with robust support, indicating a stable topology (Fig. 1). The isolates CGMCC 3.28460 and ZY 24.002, collected and described in this study, formed a well-supported single branch. *Pa.sinensis* and *Pa.chrysanthemi* were identified as sister species, constituting an independent clade with BP and BPP values of 100% and 1, respectively, with a stable topology. In addition, CGMCC 3.27537, ZY 22.079, ZY 22.080, and ZY 22.081 clustered together (BP = 100%, BPP = 1) and were closely related to *Phialoparvums. str.* (Fig. 1).

### ﻿Taxonomy

#### ﻿ Paragibellulopsis sinensis

Taxon classificationFungiGlomerellalesPlectosphaerellaceae

H. Pan & Zhi.Y. Zhang sp. nov.

38546FBB-168A-5B47-B0C6-CA2C81D51B68

856667

Fig. 2

##### Type.

China • Beijing, Xishan National Forest Park, 39.97°N, 116.20°E, soil, 4 June 2024, Zhi-Yuan Zhang (holotype HMAS 353383, dried culture; culture ex-type CGMCC 3.28460, *ibid*., ZY 24.001).

##### Etymology.

The epithet “sinensis” (Lat.) refers to China, where the species was collected.

##### Description.

***Culture characteristics*** (14 days at 25 °C): Colony on PDA 35–37 mm diam. white to grey, felty, margin ﬁmbriate; reverse: white to grey. Colony on OA 44–46 mm diam. white to grey, flat, margin entire, aerial mycelia extremely sparse; reverse: white to grey. Colony on SNA 25–30 mm diam. white, floccose, margin irregular; reverse: white.

Mycelium consisting of branched, septate, hyaline, and smooth-walled hyphae, 1–3 μm wide. ***Conidiophores*** arising from submerged or superﬁcial hyphae, erect or slanted, simple or poorly branched, often reduced to conidiogenous cells. ***Conidiogenous cells*** monophialidic, terminal, lateral, cylindrical, straight or curved, hyaline, smooth-walled, with funnel-shaped collarette and a distinct periclinal thickening at the conidiogenous locus, 20–39 μm long, 1.5–4 μm wide at the base. ***Conidia*** aggregated in small, slimy heads, hyaline, cylindrical with tapering ends, straight or slightly curved, smooth-walled, 1-celled, 5.5–9 × 2–2.5 μm (n = 50). ***Chlamydospores*** terminal, lateral, intercalary, single, or, in short, in chains, globose to ellipsoid, cylindrical, hyaline, smooth-walled, 6–10 × 5.5–7 μm (n = 50). ***Sexual morph*** unknown.

**Figure 2.**
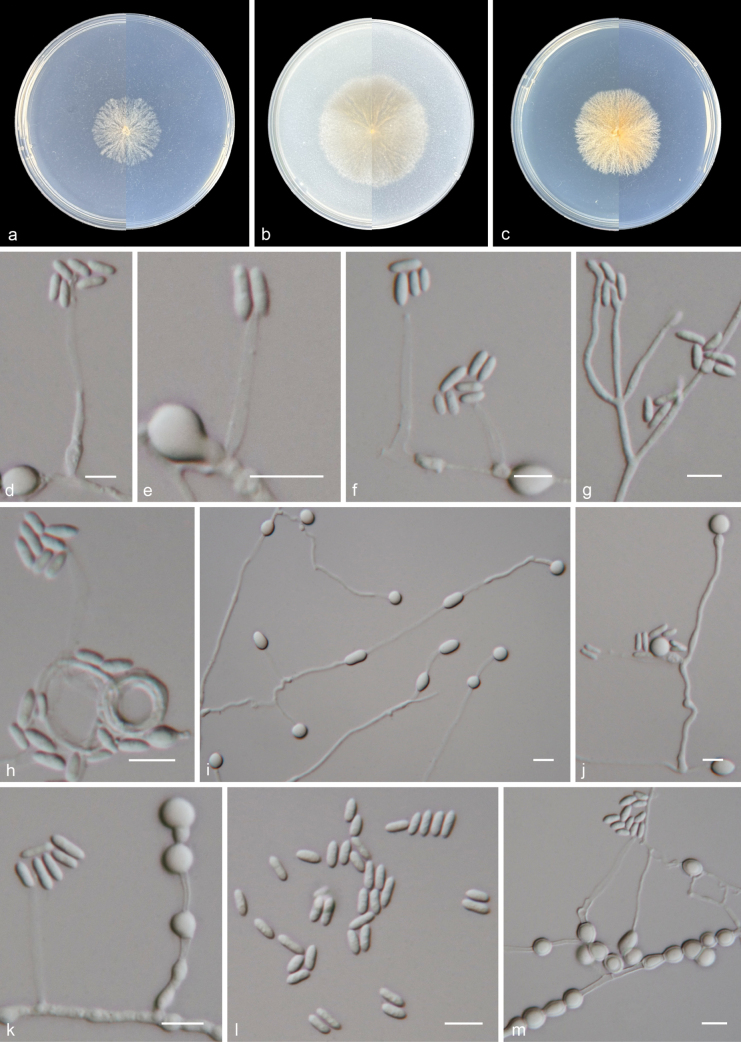
*Paragibellulopsissinensis* (from holotype HMAS 353383) **a–c** upper and reverse views of cultures on PDA, OA, and SNA after 14 days at 25 °C **d–f** conidiogenous cells **g** conidiophores **h** hyphal coil and conidiogenous cells **i, m** chlamydospores **j, k** conidia and chlamydospores **l** conidia. Scale bars: 10 μm (**d–m**).

##### Geographical distribution.

Beijing, China.

##### Additional material examined.

China • Beijing, Xishan National Forest Park, 39.97°N, 116.20°E, soil, 4 June 2022, Xin Li, ZY 24.002.

##### Notes.

Phylogenetic analysis showed that two new isolates (CGMCC 3.28460 and ZY 24.002) clustered in a single subclade with high supported value (100/1) and were nested in *Paragibellulopsiss. str.* (Fig. 1). Morphologically, *Pa.sinensis* differs from *Pa.chrysanthemi* by its short conidiogenous cells (20–39 μm in *Pa.sinensis* vs. 36–72 μm in *Pa.chrysanthemi*) and aseptate and smaller conidia (5.5–9 × 2–2.5 μm in *Pa.sinensis* vs. 12.5–15.5 × 2.7–3.7 μm in *Pa.chrysanthemi*) (Hirooka et al. 2014). In addition, *Pa.sinensis* can be distinguished from *Pa.chrysanthemi* by their low sequence similarities. In a comparison of ITS, LSU, and *tef-1α* nucleotides, *Pa.sinensis* (ex-type CGMCC 3.28460) has 97.2%, 98.5%, and 98.8% similarity in ITS (467/480 bp, four gaps), LSU (557/565 bp, no gap), and *tef-1α* (432/437 bp, no gap), which is different from *Pa.bifurcatum* (ex-type MAFF 242621).

#### ﻿ Phialoparvum sinense

Taxon classificationFungiGlomerellalesPlectosphaerellaceae

H. Pan & Zhi.Y. Zhang sp. nov.

76E2BCDA-D5FF-59BE-BCDB-4A2B06C6A042

853675

Fig. 3

##### Type.

China • Guizhou Province, Guiyang City, Xiuwen County, Zhazuo Town, Guizhou Wildlife Park, 26.85°N, 106.69°E, soil, 25 May 2022, Zhi-Yuan Zhang (holotype HMAS 353384, dried culture; culture ex-type CGMCC 3.27537, *ibid*., ZY 22.078).

##### Etymology.

The epithet “*sinense*” (Lat.) refers to China, where the species was collected.

##### Description.

***Culture characteristics*** (14 days at 25 °C): Colony on PDA 67–69 mm diam. white aluminum to grey-white from center to margin, flocculent, subcircular, margin regular; reverse: tele grey to grey-white from center to margin. Colony on OA 58–60 mm diam. white, flocculent, subcircular, margin regular; reverse: white. Colony on SNA 25–27 mm diam. white, flattened, hyphae sparse; reverse: white.

Mycelium consisting of branched, septate, hyaline, and smooth-walled hyphae, 1–3 μm wide. ***Conidiophores*** solitary, erect, arising directly from vegetative hyphae or ropes of hyphae, unbranched or poorly branched. ***Phialides*** lateral, terminal, subulate, hyaline, smooth-walled, 8.5–50.5 × 1–3 μm, with cylindrical collarette and conspicuous periclinal thickening at the conidiogenous locus; adelophialides sometimes present, up to 4 μm long; polyphialides with two conidiogenous loci are occasionally present. ***Conidia*** arranged in slimy heads, obovate with slightly obtuse base, sometimes cylindrical with slightly obtuse base, 1-celled, hyaline, smooth-walled, 2–6.5 × 1–3 μm (av. 4.5 × 2, n = 50). ***Sexual morph*** undetermined.

**Figure 3.**
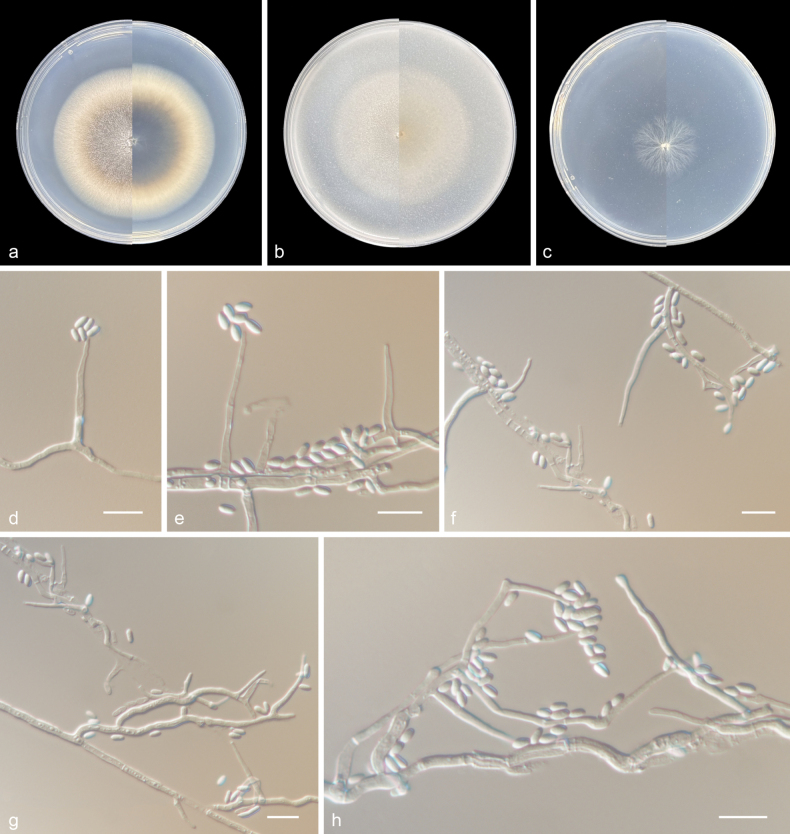
*Phialoparvumsinense* (from holotype HMAS 353384) **a–c** upper and reverse views of cultures on PDA, OA, and SNA after 14 days at 25 °C **d, e** monophialides and conidia **f** adelophialides and conidia **g, h** polyphialide and conidium. Scale bars: 10 μm (**d–h**).

##### Geographical distribution.

Guizhou Province, China.

##### Additional material examined.

China • Guizhou Province, Guiyang City, Xiuwen County, Zhazuo Town, Guizhou Wildlife Park, 26.85°N, 106.69°E, soil, 25 May 2022, Z.Y. Zhang, ZY 22.079, *ibid*., ZY 22.080, and ZY 22.081.

##### Notes.

Phylogenetic analysis showed that four new isolates (CGMCC 3.27537, ZY 22.079, ZY 22.080, and ZY 22.081) clustered in a single subclade with high support values (100/1) and were nested in *Phialoparvums. str.* (Fig. 1). Morphologically, *Ph.sinense* differs from other species in the genus *Phialoparvum* in that it produces obovate conidia and longer phialides (Giraldo and Crous 2019; Giraldo et al. 2019). In addition, they can be distinguished by their low sequence similarities. In a comparison of ITS, LSU, *rpb2*, and *tef-1α* nucleotides, *Ph.sinense* (ex-type CGMCC 3.27537) has 95.3%, 97.2%, 93.5%, and 96.3% similarity in ITS (469/492 bp, seven gaps), LSU (773/795 bp, four gaps), *rpb2* (262/280 bp, no gap), and *tef-1α* (758/787 bp, no gap), which is different from *Ph.bifurcatum* (ex-type CBS 299.70B). In a comparison of ITS, LSU, and *rpb2* nucleotides, *Ph.sinense* (CGMCC 3.27537) has 96.5%, 98.1%, and 91.9% similarity in ITS (453/469 bp, four gaps), LSU (778/793 bp, no gap), and *rpb2* (751/817 bp, no gap), which is different from *Ph.maaspleinense* (ex-type CBS 145321). In a comparison of ITS, LSU, and *rpb2* nucleotides, *Ph.sinense* (CGMCC 3.27537) has 96.6%, 98.3%, and 92.7% similarity in ITS (483/500 bp, five gaps), LSU (780/793 bp, no gap), and *rpb2* (694/748 bp, no gap), which is different from *Ph.rietveltiae* (ex-type CBS 145322).

### ﻿Key to the genus *Phialoparvum*

**Table:** 

1	Phialides subulate	**2**
–	Phialides subulate to ampulliform	**3**
2	Conidia cylindrical, occasionally with one or two guttules	***P.bifurcatum***
–	Conidia cylindrical, with slightly pointed ends	***P.rietveltiae***
3	Conidia cylindrical	***P.maaspleinense***
–	Conidia obovate	***P.sinense***

## ﻿Discussion

Currently, the plectosphaerellaceous fungi comprise more than 20 genera (Hyde et al. 2017; Tibpromma et al. 2018; Giraldo and Crous 2019; Phukhamsakda et al. 2020; Crous et al. 2023a; Hou et al. 2023). They exhibited a wide distribution and were commonly observed on various substrates or hosts, including soil, plants, fungi, humans, decayed wood, bark, and leaves (Hyde et al. 2014; Giraldo and Crous 2019). While conducting taxonomic studies of genera within Plectosphaerellaceae based on multi-locus phylogenetic and morphological evidence, Giraldo and Crous (2019) transferred *Gibellulopsischrysanthemi* from *Gibellulopsis* and established it as the type species of the newly erected genus *Paragibellulopsis*. To date, *Paragibellulopsis* includes only one species (*Pa.chrysanthemi*), which was isolated from rotten leaves of Chrysanthemumcoronariumvar.spatiosum (Hirooka et al. 2014). The novel species *Pa.sinensis* proposed in this study was isolated from soil, which expands our understanding of the trophic modes within this genus. Furthermore, Giraldo and Crous (2019) established the genus *Phialoparvum* based on *Ph.bifurcatum* as the type species, which was isolated from soil. Subsequently, two other valid species of the genus *Phialoparvum* (*Ph.maaspleinense* and *Ph.rietveltiae*) were reported, which were isolated from Dutch garden soil (Giraldo et al. 2019). All three species were isolated from Europe, whereas *Ph.sinense* was obtained from China, suggesting this genus may have a wide distribution. This study enhances our understanding of species diversity and geographical distribution within these two genera.

The macroscopic and microscopic morphology of most species in the plectosphaerellaceous fungi are quite similar, and it is difficult to distinguish specific species based on only morphological features. Thus, it is often necessary to combine morphological and molecular data for species identification. According to the new species delimitation criteria proposed by Jeewon and Hyde (2016), phylogenetic analyses should incorporate the ITS regions and at least one protein gene, with a minimum of >1.5% nucleotide differences in the ITS regions between closely related species. In this study, two novel species, *Pa.sinensis* and *Ph.sinense*, were identified and characterized through meticulous morphological examination, rigorous phylogenetic analyses, and nucleotide divergence assessments among closely related species.

It was observed that a prominent characteristic of species within the *Paragibellulopsis* genus was the monophialidic of conidiogenous cells, wherein conidia typically produced in slimy heads, cylindrical, straight, or slightly curved (Hirooka et al. 2014; Giraldo and Crous 2019). The primary distinguishing feature of *Phialoparvum* species lies in phialides enteroblastic, mono- and polyphialidic; conidia cylindrical, 1-celled, arranged in slimy heads (Giraldo and Crous 2019). The conidiogenous cells of *Pa.sinensis* collected in this study were monophialidic; the conidia aggregated in small, slimy heads, cylindrical, straight, or slightly curved. These characteristics aligned closely with the primary identification features described for *Paragibellulopsis* species by Giraldo and Crous (2019). However, *Pa.sinensis* differs primarily from *Pa.chrysanthemi* in producing aseptate and comparatively smaller conidia. The phialides of *Ph.sinense* mono- and polyphialidic; conidia obovate, sometimes cylindrical, arranged in slimy heads, which was consistent with previous studies on *Phialoparvum* species (Giraldo et al. 2019; Giraldo and Crous 2019). *Ph.sinense* differs markedly from other *Phialoparvum* species by producing obovoid conidia and conspicuously longer phialides.

## Supplementary Material

XML Treatment for Paragibellulopsis sinensis

XML Treatment for Phialoparvum sinense

## ﻿Additional information

### Author ORCIDs

Heng Pan https://orcid.org/0009-0003-7472-3199

Shuo-Qiu Tong https://orcid.org/0009-0006-9422-5551

Zeng Chen https://orcid.org/0009-0009-8437-2170

Yun-Jie Wu https://orcid.org/0009-0005-1316-5546

Yi Wang https://orcid.org/0000-0002-6265-3571

Gang Tao https://orcid.org/0000-0002-0882-3752

Zhi-Yuan Zhang https://orcid.org/0000-0003-2031-7518

### Data availability

All of the data that support the findings of this study are available in the main text.

## References

[B1] Chen Q Jiang JR Zhang GZ Cai L Crous PW (2015) Resolving the *Phoma* enigma. Studies in Mycology 82: 137–217. 10.1016/j.simyco.2015.10.003 26955202 PMC4774273

[B2] Crous PW Lombard L Sandoval-Denis M Seifert KA Schroers HJ Chaverri P Gené J Guarro J Hirooka Y Bensch K Kema GHJ Lamprecht SC Cai L Rossman AY Stadler M Summerbell RC Taylor JW Ploch S Visagie CM Yilmaz N Frisvad JC Abdel-Azeem AM Abdollahzadeh J Abdolrasouli A Akulov A Alberts JF Araújo JPM Ariyawansa HA Bakhshi M Bendiksby M Ben Hadj Amor A Bezerra JDP Boekhout T Câmara MPS Carbia M Cardinali G Castañeda-Ruiz RF Celis A Chaturvedi V Collemare J Croll D Damm U Decock CA de Vries RP Ezekiel CN Fan XL Fernández NB Gaya E González CD Gramaje D Groenewald JZ Grube M Guevara-Suarez M Gupta VK Guarnaccia V Haddaji A Hagen F Haelewaters D Hansen K Hashimoto A Hernández-Restrepo M Houbraken J Hubka V Hyde KD Iturriaga T Jeewon R Johnston PR Jurjević Ž Karalti İ Korsten L Kuramae EE Kušan I Labuda R Lawrence DP Lee HB Lechat C Li HY Litovka YA Maharachchikumbura SSN Marin-Felix Y Matio Kemkuignou B Matočec N McTaggart AR Mlčoch P Mugnai L Nakashima C Nilsson RH Noumeur SR Pavlov IN Peralta MP Phillips AJL Pitt JI Polizzi G Quaedvlieg W Rajeshkumar KC Restrepo S Rhaiem A Robert J Robert V Rodrigues AM Salgado-Salazar C Samson RA Santos ACS Shivas RG Souza-Motta CM Sun GY Swart WJ Szoke S Tan YP Taylor JE Taylor PWJ Tiago PV Váczy KZ van de Wiele N van der Merwe NA Verkley GJM Vieira WAS Vizzini A Weir BS Wijayawardene NN Xia JW Yáñez-Morales MJ Yurkov A Zamora JC Zare R Zhang CL Thines M (2021) *Fusarium*: More than a node or a foot-shaped basal cell. Studies in Mycology 98: 1–184. 10.1016/j.simyco.2021.100116 PMC8379525 34466168

[B3] Crous PW Osieck ER Shivas RG Tan YP Bishop-Hurley SL Esteve-Raventós F Larsson E Luangsa-ard JJ Pancorbo F Balashov S Baseia IG Boekhout T Chandranayaka S Cowan DA Cruz RHSF Czachura P De la Peña-Lastra S Dovana F Drury B Fell J Flakus A Fotedar R Jurjević Ž Kolecka A Mack J Maggs-Kölling G Mahadevakumar S Mateos A Mongkolsamrit S Noisripoom W Plaza M Overy DP Piątek M Sandoval-Denis M Vauras J Wingfield MJ Abell SE Ahmadpour A Akulov A Alavi F Alavi Z Altés A Alvarado P Anand G Ashtekar N Assyov B Banc-Prandi G Barbosa KD Barreto GG Bellanger JM Bezerra JL Bhat DJ Bilański P Bose T Bozok F Chaves J Costa-Rezende DH Danteswari C Darmostuk V Delgado G Denman S Eichmeier A Etayo J Eyssartier G Faulwetter S Ganga KGG Ghosta Y Goh J Góis JS Gramaje D Granit L Groenewald M Gulden G Gusmão LFP Hammerbacher A Heidarian Z Hywel-Jones N Jankowiak R Kaliyaperumal M Kaygusuz O Kezo K Khonsanit A Kumar S Kuo CH Læssøe T Latha KPD Loizides M Luo SM Maciá-Vicente JG Manimohan P Marbach PAS Marinho P Marney TS Marques G Martín MP Miller AN Mondello F Moreno G Mufeeda KT Mun HY Nau T Nkomo T Okrasińska A Oliveira JPAF Oliveira RL Ortiz DA Pawłowska J Pérez-De-Gregorio MÀ Podile AR Portugal A Privitera N Rajeshkumar KC Rauf I Rian B Rigueiro-Rodríguez A Rivas-Torres GF Rodriguez-Flakus P Romero-Gordillo M Saar I Saba M Santos CD Sarma PVSRN Siquier JL Sleiman S Spetik M Sridhar KR Stryjak-Bogacka M Szczepańska K Taşkın H Tennakoon DS Thanakitpipattana D Trovão J Türkekul İ van Iperen AL van ’t Hof P Vasquez G Visagie CM Wingfield BD Wong PTW Yang WX Yarar M Yarden O Yilmaz N Zhang N Zhu YN Groenewald JZ (2023a) Fungal Planet description sheets: 1478–1549. Persoonia 50(1): 158–310. 10.3767/persoonia.2023.50.05 38567263 PMC10983837

[B4] Crous PW Akulov A Balashov S Boers J Braun U Castillo J Delgado MA Denman S Erhard A Gusella G Jurjević Ž Kruse J Malloch DW Osieck ER Polizzi G Schumacher RK Slootweg E Starink-Willemse M van Iperen AL Verkley GJM Groenewald JZ (2023b) New and interesting Fungi. 6. Fungal Systematics and Evolution 11(1): 109–156. 10.3114/fuse.2023.11.09 38545457 PMC10966675

[B5] Domsch KH Gams W Anderson TH (2007) Compendium of soil fungi, 2^nd^ edition. IHW Verlag Publishing, Eching, Germany.

[B6] Drummond A Rambaut A (2007) BEAST: Bayesian evolutionary analysis by sampling trees. BMC Evolutionary Biology 7: 214. 10.1186/1471-2148-7-214 PMC2247476 17996036

[B7] Gams W (1971) Cephalosporium-artige Schimmelpilz*e* (Hyphomycetes). Gustav Fischer Verlag, Stuttgart, Germany.

[B8] Gams W (1975) *Cephalosporium*-like Hyphomycetes: Some tropical species. Transactions of the British Mycological Society 64: 389–404. 10.1016/S0007-1536(75)80138-0

[B9] Giraldo A Crous PW (2019) Inside Plectosphaerellaceae. Studies in Mycology 92(1): 227–286. 10.1016/j.simyco.2018.10.005 30518989 PMC6276054

[B10] Giraldo A Hernández-Restrepo M Crous PW (2019) New plectosphaerellaceous species from dutch garden soil. Mycological Progress 18(9): 1135–1154. 10.1007/s11557-019-01511-4

[B11] Hirooka Y Kawaradani M Sato T (2014) Description of *Gibellulopsischrysanthemi* sp. nov. from leaves of garland chrysanthemum. Mycological Progress 13: 13–19. 10.1007/s11557-012-0887-x

[B12] Hou LW Giraldo A Groenewald JZ Rämä T Summerbell RC Huang GZ Cai L Crous PW (2023) Redisposition of acremonium-like fungi in Hypocreales. Studies in Mycology 105: 23–203. 10.3114/sim.2023.105.02 38895703 PMC11182610

[B13] Hyde KD Nilsson RH Alias SA Ariyawansa HA Blair JE Cai L de Cock AWAM Dissanayake AJ Glockling SL Goonasekara ID Gorczak M Hahn M Jayawardena RS van Kan JAL Laurence MH Lévesque CA Li XH Liu JK Maharachchikumbura SSN Manamgoda DS Martin FN McKenzie EHC McTaggart AR Mortimer PE Nair PVR Pawłowska J Rintoul TL Shivas RG Spies CFJ Summerell BA Taylor PWJ Terhem RB Udayanga D Vaghefi N Walther G Wilk M Wrzosek M Xu JC Yan JY Zhou N (2014) One stop shop: backbones trees for important pytopathogenic genera: I (2014). Fungal Diversity 67(1): 21–125. 10.1007/s13225-014-0298-1

[B14] Hyde KD Norphanphoun C Abreu VP Bazzicalupo A Thilini Chethana KW Clericuzio M Dayarathne MC Dissanayake AJ Ekanayaka AH He MQ Hongsanan S Huang SK Jayasiri SC Jayawardena RS Karunarathna A Konta S Kušan I Lee H Li J Lin CG Liu NG Lu YZ Luo ZL Manawasinghe IS Mapook A Perera RH Phookamsak R Phukhamsakda C Siedlecki I Soares AM Tennakoon DS Tian Q Tibpromma S Wanasinghe DN Xiao YP Yang J Zeng XY Abdel-Aziz FA Li WJ Senanayake IC Shang QJ Daranagama DA de Silva NI Thambugala KM Abdel-Wahab MA Bahkali AH Berbee ML Boonmee S Bhat DJ Bulgakov TS Buyck B Camporesi E Castañeda-Ruiz RF Chomnunti P Doilom M Dovana F Gibertoni TB Jadan M Jeewon R Jones EBG Kang JC Karunarathna SC Lim YW Liu J-K Liu ZY Plautz Jr HL Lumyong S Maharachchikumbura SSN Matočec N McKenzie EHC Mešić A Miller D Pawłowska J Pereira OL Promputtha I Romero AI Ryvarden L Su HY Suetrong S Tkalčec Z Vizzini A Wen TC Wisitrassameewong K Wrzosek M Xu JC Zhao Q Zhao RL Mortimer PE (2017) Fungal diversity notes 603–708: Taxonomic and phylogenetic notes on genera and species. Fungal Diversity 87(1): 1–235. 10.1007/s13225-017-0391-3

[B15] Hyde KD Noorabadi MT Thiyagaraja V He MQ Johnston PR Wijesinghe SN Armand A Biketova AY Chethana KWT Erdoğdu M Ge ZW Groenewald JZ Hongsanan S Kušan I Leontyev DV Li DW Lin CG Liu NG Maharachchikumbura SSN Matočec N May TW McKenzie EHC Mešić A Perera RH Phukhamsakda C Piątek M Samarakoon MC Selcuk F Senanayake IC Tanney JB Tian Q Vizzini A Wanasinghe DN Wannasawang N Wijayawardene NN Zhao RL Abdel-Wahab MA Abdollahzadeh J Abeywickrama PD Abhinav Absalan S Acharya K Afshari N Afshan NS Afzalinia S Ahmadpour SA Akulov O Alizadeh A Alizadeh M Al-Sadi AM Alves A Alves VCS Alves-Silva G Antonín V Aouali S Aptroot A Apurillo CCS Arias RM Asgari B Asghari R Assis DMA Assyov B Atienza V Aumentado HDR Avasthi S Azevedo E Bakhshi M Bao DF Baral HO Barata M Barbosa KD Barbosa RN Barbosa FR Baroncelli R Barreto GG Baschien C Bennett RM Bera I Bezerra JDP Bhunjun CS Bianchinotti MV Błaszkowski J Boekhout T Bonito GM Boonmee S Boonyuen N Bortnikov FM Bregant C Bundhun D Burgaud G Buyck B Caeiro MF Cabarroi-Hernández M Cai M Feng Cai L Calabon MS Calaça FJS Callalli M Câmara MPS Cano-Lira J Cao B Carlavilla JR Carvalho A Carvalho TG Castañeda-Ruiz RF Catania MDV Cazabonne J Cedeño-Sanchez M Chaharmiri-Dokhaharani S Chaiwan N Chakraborty N Cheewankoon R Chen C Chen J Chen Q Chen YP Chinaglia S Coelho-Nascimento CC Coleine C CostaRezende DH Cortés-Pérez A Crouch JA Crous PW Cruz RHSF Czachura P Damm U Darmostuk V Daroodi Z Das K Das K Davoodian N Davydov EA da Silva GA da IR da Silva Silva RMF da Silva Santos AC Dai DQ Dai YC de Groot Michiel D De Kesel A De Lange R de Medeiros EV de Souza CFA de Souza FA dela Cruz TEE Decock C Delgado G Denchev CM Denchev TT Deng YL Dentinger BTM Devadatha B Dianese JC Dima B Doilom M Dissanayake AJ Dissanayake DMLS Dissanayake LS Diniz AG Dolatabadi S Dong JH Dong W Dong ZY Drechsler-Santos ER Druzhinina IS Du TY Dubey MK Dutta AK Elliott TF Elshahed MS Egidi E Eisvand P Fan L Fan X Fan XL Fedosova AG Ferro LO Fiuza PO Flakus A W. Fonseca EO Fryar SC Gabaldón T Gajanayake AJ Gannibal PB Gao F GarcíaSánchez D García-Sandoval R Garrido-Benavent I Garzoli L Gasca-Pineda J Gautam AK Gené J Ghobad-Nejhad M Ghosh A Giachini AJ Gibertoni TB Gentekaki E Gmoshinskiy VI GóesNeto A Gomdola D Gorjón SP Goto BT Granados-Montero MM Griffith GW Groenewald M Grossart H-P Gu ZR Gueidan C Gunarathne A Gunaseelan S Guo SL Gusmão LFP Gutierrez AC Guzmán-Dávalos L Haelewaters D Haituk H Halling RE He SC Heredia G HernándezRestrepo M Hosoya T Hoog SD Horak E Hou CL Houbraken J Htet ZH Huang SK Huang WJ Hurdeal VG Hustad VP Inácio CA Janik P Jayalal RGU Jayasiri SC Jayawardena RS Jeewon R Jerônimo GH Jin J Jones EBG Joshi Y Jurjević Ž Justo A Kakishima M Kaliyaperumal M Kang GP Kang JC Karimi O Karunarathna SC Karpov SA Kezo K Khalid AN Khan MK Khuna S Khyaju S Kirchmair M Klawonn I Kraisitudomsook N Kukwa M Kularathnage ND Kumar S Lachance MA Lado C Latha KPD Lee HB Leonardi M Lestari AS Li C Li H. Li J Li Q Li Y Li YC Li YX Liao CF Lima JLR Lima JMS Lima NB Lin L Linaldeddu BT Linn MM Liu F Liu JK Liu JW Liu S Liu SL Liu XF Liu XY Longcore JE Luangharn T Luangsa-ard JJ Lu L Lu YZ Lumbsch HT Luo L Luo M Luo ZL Ma J Madagammana AD Madhushan A Madrid H Magurno F Magyar D Mahadevakumar S Malosso E Malysh JM Mamarabadi M Manawasinghe IS Manfrino RG Manimohan P Mao N Mapook A Marchese P Marasinghe DS Mardones M Marin-Felix Y Masigol H Mehrabi M MehrabiKoushki M Meiras-Ottoni A de Melo RFR Mendes-Alvarenga RL Mendieta S Meng QF Menkis A Menolli Jr N Mikšík M Miller SL Moncada B Moncalvo JM Monteiro JS Monteiro M Mora-Montes HM Moroz EL Moura JC Muhammad U Mukhopadhyay S Nagy GL Najam ul Sehar A Najafiniya M Nanayakkara CM Naseer A Nascimento ECR Nascimento SS Neuhauser S Neves MA Niazi AR Nie Yong Nilsson RH Nogueira PTS Novozhilov YK Noordeloos M Norphanphoun C Nuñez Otaño N O’Donnell RP Oehl F Oliveira JA Oliveira Junior I Oliveira NVL Oliveira PHF Orihara T Oset M Pang KL Papp V Pathirana LS Peintner U Pem D Pereira OL Pérez-Moreno J Pérez-Ortega S Péter G Pires-Zottarelli CLA Phonemany M Phongeun S Pošta A Prazeres JFSA Quan Y Quandt CA Queiroz MB Radek R Rahnama K Raj KNA Rajeshkumar KC Rajwar Soumyadeep Ralaiveloarisoa AB Rämä T Ramírez-Cruz V Rambold G Rathnayaka AR Raza M Ren GC Rinaldi AC Rivas-Ferreiro M Robledo GL Ronikier A Rossi W Rusevska K Ryberg M Safi A Salimi F Salvador-Montoya CA Samant B Samaradiwakara NP Sánchez-Castro I Sandoval-Denis M Santiago ALCMA Santos ACDS Santos LA dos Sarma VV Sarwar S. Savchenko A Savchenko K Saxena RK Schoutteten N Selbmann L Ševčíková H Sharma A Shen HW Shen YM Shu YX Silva HF Silva-Filho AGS Silva VSH Simmons DR Singh R Sir EB Sohrabi M Souza FA Souza-Motta CM Sriindrasutdhi V Sruthi OP Stadler M Stemler J Stephenson SL Stoyneva-Gaertner MP Strassert JFH Stryjak-Bogacka M Su H Sun YR Svantesson S Sysouphanthong P Takamatsu S Tan TH Tanaka K Tang C Tang X Taylor JE Taylor PWJ Tennakoon DS Thakshila SAD Thambugala KM Thamodini GK Thilanga D Thines M Tiago PV Tian XG Tian WH Tibpromma S Tkalčec Z Tokarev YS Tomšovský M Torruella G Tsurykau A Udayanga D Ulukapı M Untereiner WA Usman M Uzunov BA Vadthanarat S Valenzuela R Van den Wyngaert S Van Vooren N Velez P Verma RK Vieira LC Vieira WAS Vinzelj JM Tang AMC Walker A Walker AK Wang QM Wang Y Wang XY Wang ZY Wannathes N Wartchow F Weerakoon G Wei DP Wei X White JF Wijesundara DSA Wisitrassameewong K Worobiec G Wu HX Wu N Xiong YR Xu B Xu JP Xu R Xu RF Xu RJ Yadav S Yakovchenko LS Yang HD Yang X Yang YH Yang Y Yang YY Yoshioka R Youssef Noha H Yu FM Yu ZF Yuan LL Yuan Q Zabin DA Zamora JC Zapata CV Zare R Zeng M Zeng XY Zhang JF Zhang JY Zhang S Zhang XC Zhao CL Zhao H Zhao Q Zhao H Zhao HJ Zhou HM Zhu XY Zmitrovich IV Zucconi L Zvyagina E (2024) The 2024 Outline of Fungi and fungus-like taxa. Mycosphere: Journal of Fungal Biology 15(1): 5146–6239. 10.5943/mycosphere/15/1/25

[B16] Jeewon R Hyde KD (2016) Establishing species boundaries and new taxa among fungi: Recommendations to resolve taxonomic ambiguities. Mycosphere: Journal of Fungal Biology 7(11): 1669–1677. 10.5943/mycosphere/7/11/4

[B17] Kalyaanamoorthy S Minh BQ Wong TKF Von HA Jermiin LS (2017) ModelFinder: Fast model selection for accurate phylogenetic estimates. Nature Methods 14(6): 587–589. 10.1038/nmeth.4285 28481363 PMC5453245

[B18] Katoh K Standley DM (2013) MAFFT multiple sequence alignment software version 7: Improvements in performance and usability. Molecular Biology and Evolution 30(4): 772–780. 10.1093/molbev/mst010 23329690 PMC3603318

[B19] Liu YJ Whelen S Hall BD (1999) Phylogenetic relationships among ascomycetes: Evidence from an RNA polymerase II subunit. Molecular Biology and Evolution 16(12): 1799–1808. 10.1093/oxfordjournals.molbev.a026092 10605121

[B20] Minh BQ Nguyen MAT Von HA (2013) Ultrafast approximation for phylogenetic bootstrap. Molecular Biology and Evolution 30(5): 1188–1195. 10.1093/molbev/mst024 23418397 PMC3670741

[B21] Nguyen LT Schmidt HA von Haeseler A Minh BQ (2015) IQ-TREE: A fast and effective stochastic algorithm for estimating maximum-likelihood phylogenies. Molecular Biology and Evolution 32(1): 268–274. 10.1093/molbev/msu300 25371430 PMC4271533

[B22] Perdomo H Sutton DA García D Fothergill AW Cano J Gené J Summerbell RC Rinaldi MG Guarro J (2011) Spectrum of clinically relevant *Acremonium* species in the United States. Journal of Clinical Microbiology 49: 243–256. 10.1128/jcm.00793-10 21068274 PMC3020405

[B23] Phukhamsakda C McKenzie EHC Phillips AJL Jones EBG Bhat DJ Stadler M Bhunjun CS Wanasinghe DN Thongbai B Camporesi E Ertz D Jayawardena RS Perera RH Ekanayake AH Tibpromma S Doilom M Xu JC Hyde KD (2020) Microfungi associated with *Clematis* (Ranunculaceae) with an integrated approach to delimiting species boundaries. Fungal Diversity 102(1): 1–203. 10.1007/s13225-020-00448-4

[B24] Rehner SA Buckley E (2005) A Beauveria phylogeny inferred from nuclear ITS and EF1-α sequences: Evidence for cryptic diversification and links to Cordyceps teleomorphs. Mycologia 97(1): 84–98. 10.3852/mycologia.97.1.84 16389960

[B25] Ronquist F Teslenko M van der Mark P Ayres DL Darling A Höhna S Larget B Liu L Suchard MA Huelsenbeck JP (2012) MrBayes 3.2: Efficient Bayesian phylogenetic inference and model choice across a large model space. Systematic Biology 61(3): 539–542. 10.1093/sysbio/sys029 22357727 PMC3329765

[B26] Senanayake IC Rathnayaka AR Marasinghe DS Calabon MS Gentekaki E Lee HB Hurdeal VG Pem D Dissanayake LS Wijesinghe SN Bundhun D Nguyen TTT Goonasekara ID Abeywickrama PD Bhunjun CS Jayawardena RS Wanasinghe DN Jeewon R Bhat DJ Xiang MM (2020) Morphological approaches in studying fungi: Collection, examination, isolation, sporulation and preservation. Mycosphere: Journal of Fungal Biology 11: 2678–2754. 10.5943/mycosphere/11/1/20

[B27] Summerbell RC Gueidan C Schroers HJ de Hoog GS Starink M Arocha Rosete Y Guarro J Scott JA (2011) *Acremonium* phylogenetic overview and revision of *Gliomastix*, *Sarocladium*, and *Trichothecium*. Studies in Mycology 68: 139–162. 10.3114/sim.2011.68.06 PMC3065988 21523192

[B28] Tamura K Stecher G Peterson D Filipski A Kumar S (2013) MEGA6: Molecular evolutionary genetics analysis version 6.0. Molecular Biology and Evolution 30(12): 2725–2729. 10.1093/molbev/mst197 24132122 PMC3840312

[B29] Tibpromma S Hyde KD McKenzie EHC Bhat DJ Phillips AJL Wanasinghe DN Samarakoon MC Jayawardena RS Dissanayake AJ Tennakoon DS Doilom M Phookamsak R Tang AMC Xu J Mortimer PE Promputtha I Maharachchikumbura SSN Khan S Karunarathna SC (2018) Fungal diversity notes 840–928: Micro-fungi associated with Pandanaceae. Fungal Diversity 93: 1–160. 10.1007/s13225-018-0408-6

[B30] Verkley GJM Quaedvlieg W Shin H-D Crous PW (2013) A new approach to species delimitation in *Septoria*. Studies in Mycology 75: 213–305. 10.3114/sim0018 PMC3713889 24014901

[B31] Vilgalys R Hester M (1990) Rapid genetic identification and mapping of enzymatically amplified ribosomal DNA from several *Cryptococcus* species. Journal of Bacteriology 172(8): 4238–4246. 10.1128/jb.172.8.4238-4246.1990 2376561 PMC213247

[B32] White TJ Bruns T Lee SJWT Taylor J (1990) Amplification and direct sequencing of fungal ribosomal RNA genes for phylogenetics. PCR protocols: a guide to methods and applications 18: 315–322. 10.1016/B978-0-12-372180-8.50042-1

[B33] Xiang CY Gao F Jakovlić I Lei HP Hu Ye Zhang H Zou H Wang GT Zhang D (2023) Using PhyloSuite for molecular phylogeny and tree‐based analyses. iMeta 2(1): e87. 10.1002/imt2.87 PMC10989932 38868339

[B34] Yeh Y-H Huang Y-M Kirschner R (2024) Plectosphaerellaceous fungi (Ascomycota) on leaf litter of the subtropical giant fern *Angiopterislygodiifolia* (Marattiaceae) in Taiwan. Nova Hedwigia 119(3–4): 419–448. 10.1127/nova_hedwigia/2024/0960

[B35] Zare R Gams W Starink-Willemse M Summerbell RC (2007) *Gibellulopsis*, a suitable genus for *Verticilliumnigrescens*, and *Musicillium*, a new genus for *V.theobromae*. Nova Hedwigia 85(3–4): 463–489. 10.1127/0029-5035/2007/0085-0463

[B36] Zhang ZY Li X Chen WH Liang JD Han YF (2023) Culturable fungi from urban soils in China II, with the description of 18 novel species in Ascomycota (Dothideomycetes, Eurotiomycetes, Leotiomycetes and Sordariomycetes). MycoKeys 98: 167–220. 10.3897/mycokeys.98.102816 37425100 PMC10326621

[B37] Zhang ZY Pan H Tao G Li X Han YF Feng Y Tong SQ Ding CY (2024) Culturable mycobiota from Guizhou Wildlife Park in China. Mycosphere: Journal of Fungal Biology 15(1): 654–763. 10.5943/mycosphere/15/1/5

